# Inhibitory effects of retinoic acid metabolism blocking agents (RAMBAs) on the growth of human prostate cancer cells and LNCaP prostate tumour xenografts in SCID mice

**DOI:** 10.1038/sj.bjc.6602971

**Published:** 2006-01-31

**Authors:** C K Huynh, A M H Brodie, V C O Njar

**Affiliations:** 1 Program in Toxicology, University of Maryland School of Medicine, 10 South Pine Street, MSTF 7-34F, Baltimore, MD 21201-1559, USA; 2 Department of Pharmacology and Experimental Therapeutics, University of Maryland School of Medicine, 685 West Baltimore Street, Baltimore, MD 21201-1559, USA; 3 The University of Maryland Marlene and Stewart Greenebaum Cancer Center, School of Medicine, Baltimore, MD 21201-1559, USA

**Keywords:** all-*trans*-retinoic acid (ATRA), catabolism, CYP26, inhibitors, RAMBAs, azolyl retinoids

## Abstract

In recent studies, we have identified several highly potent all-*trans*-retinoic acid (ATRA) metabolism blocking agents (RAMBAs). On the basis of previous effects of liarozole (a first-generation RAMBA) on the catabolism of ATRA and on growth of rat Dunning R3227G prostate tumours, we assessed the effects of our novel RAMBAs on human prostate tumour (PCA) cell lines. We examined three different PCA cell lines to determine their capacity to induce P450-mediated oxidation of ATRA. Among the three different cell lines, enhanced catabolism was detected in LNCaP, whereas it was not found in PC-3 and DU-145. This catabolism was strongly inhibited by our RAMBAs, the most potent being VN/14-1, VN/50-1, VN/66-1, and VN/69-1 with IC_50_ values of 6.5, 90.0, 62.5, and 90.0 nM, respectively. The RAMBAs inhibited the growth of LNCaP cells with IC_50_ values in the *μ*M-range. In LNCaP cell proliferation assays, VN/14-1, VN/50-1, VN/66-1, and VN/69-1 also enhanced by 47-, 60-, 70-, and 65-fold, respectively, the ATRA-mediated antiproliferative activity. We then examined the molecular mechanism underlying the growth inhibitory properties of ATRA alone and in combination with RAMBAs. The mechanism appeared to involve the induction of differentiation, cell-cycle arrest, and induction of apoptosis (TUNEL), involving increase in Bad expression and decrease in Bcl-2 expression. Treatment of LNCaP tumours growing in SCID mice with VN/66-1 and VN/69-1 resulted in modest but statistically significant tumour growth inhibition of 44 and 47%, respectively, while treatment with VN/14-1 was unexpectedly ineffective. These results suggest that some of our novel RAMBAs may be useful agents for the treatment of prostate cancer.

Retinoids, including vitamin A and its natural and synthetic analogues, have been used as experimental treatments in cancer patients for the last 30 years (Smith *et al*, 1992a; Sporn *et al*, 1994; Lotan, 1996). Retinoic acids play a role in proliferation, differentiation, metabolism, reproduction, and morphogenesis (De Luca *et al*, 1995; Miller, 1998). Additionally, one such natural analogue, all-*trans*-retinoic acid (ATRA), has been used successfully in the treatment of acute promyelocytic leukemia (APL) (Huang *et al*, 1988; Smith *et al*, 1992b). ATRA acts by binding to nuclear retinoic acid receptors (RARs), of which there are three subtypes, RAR-*α*, -*β*, and -*γ*. (Chambon *et al*, 1991; Mangelsdorf *et al*, 1994).

There are numerous evidence for the role of the retinoids, including ATRA, in prostate cancer (PCA). Human, animal, and epidemiological studies suggest that retinoid deficiency plays a role in PCA development (Lasnitzki and Goodman, 1974; Hayes *et al*, 1988; Carter *et al*, 1990; Reichman *et al*, 1990; Hanchette and Schwartz, 1992; Peehl *et al*, 1993). Pasquali *et al* demonstrated that levels of endogenous ATRA are five to eight times lower in human PCA tissues than in normal prostate tissues (Pasquali *et al*, 1996). Nagy *et al* (1996) demonstrated that the expression of Bcl-2, an antiapoptotic protein, was regulated by ATRA. Expression of Bcl-2 is also associated with the development of androgen resistance and invasiveness of androgen-independent PCA (McDonnell *et al*, 1992; Raffo *et al*, 1995; McDonnell *et al*, 1997). In addition, other groups have demonstrated the role of ATRA and its synthetic derivatives on growth of both the prostate gland and PCA cell lines (Pollard and Luckert, 1991; Pienta *et al*, 1993; Young *et al*, 1994; Gao *et al*, 1999).

However, the therapeutic effects of the retinoids are limited owing to their rapid *in vivo* metabolism (Muindi *et al*, 1992). The first step in the metabolism of ATRA is catalysed by CYP26A1, a cytochrome P450 enzyme, where the C-4 of ATRA is hydroxylated to 4-hydroxy-ATRA, which can then be oxidized to 4-oxo-ATRA, which undergoes further reactions to yield more polar metabolites (Frolik *et al*, 1979). CYP26 family appears to be the most dedicated ATRA 4-hydroxylase (Njar, 2002, 2006; Marill *et al*, 2003). Other CYPs, including CYP1A1, CYP1A2, CYP2C8, CYP2C9, CYP3A4, and CYP3A7 are ATRA 4-hydroxylases and are involved in ATRA metabolism as well, although their specificity for ATRA is low (Njar, 2002, 2006; Marill *et al*, 2003). PCA cells may develop resistance to ATRA using this mechanism as demonstrated by APL patients who relapse within 3–15 months after first remission with ATRA therapy (Norum, 1993).

Although research to date has concentrated on the use of exogenous retinoids, a potential new approach to the treatment and prevention of cancer is the use of retinoic acid metabolism blocking agents (RAMBAs), which increase levels of retinoic acid within tumour cells by blocking their metabolism (Wouters, 1994; Miller, 1998; Njar, 2002, 2006). Additionally, it has been shown that certain retinoids, including ATRA, are capable of directing neoplastic cells to the normal phenotype of morphological maturation and loss of proliferative capacity, thereby reversing or suppressing developing lesions and preventing cancer invasion (Sporn *et al*, 1976; Sporn, 1991; Lippman *et al*, 1994; Moon *et al*, 1994; Lotan, 1996).

We have designed and synthesized a number of novel RAMBAs to inhibit CYP26 and the other ATRA 4-hydroxylases with the goal of preventing *in vivo* metabolism and thereby increasing the endogenous levels of ATRA (Njar *et al*, 2000; Patel *et al*, 2004). These novel RAMBAs have been described as atypical, owing to their multiple biological activities especially in MCF-7 and T47D human breast cancer cells (Patel *et al*, 2004). The effects of the RAMBAs (VN/14-1, VN/50-1, VN/66-1, and VN/69-1; Figure 1), alone and in combination with ATRA, on ATRA metabolism, and on PCA cell viability, apoptosis, cell cycle, and differentiation, and on *in vivo* antitumour studies have been examined. In addition, the molecular mechanisms underlying the biological activities of these agents were also investigated. These studies are the basis of this report.

## MATERIALS AND METHODS

### Drug preparations

We have previously published the syntheses of the RAMBAs used in this study (Patel *et al*, 2004). *All*-*trans*-retinoic acid (ATRA) and the RAMBAs were dissolved in 95% ethanol and stored at −20°C in the dark. The concentrations (1 and 5 *μ*M) of the various agents (retinoids and RAMBAs) are typical for retinoid cancer cells inhibitory studies (Wu *et al*, 2001). All chemicals were purchased from Sigma Chemical Co. (St Louis, MO, USA) unless otherwise noted.

### Cell culture

Prostate cancer cell lines, LNCaP, PC3, and DU145, were incubated in the RPMI 1640 medium (Gibco-Invitrogen, Carlsbad, CA, USA) preparation containing 10% FBS (Hyclone, Logan, UT, USA) and 1% penicillin–streptomycin solution (Gibco-Invitrogen, Carlsbad, CA, USA) at 37°C and 5% CO_2_. All cells were subcultured weekly. LNCaP cells used in the following studies were performed between passages 7–20.

### Preparation of cellular CYP26 microsomes

The procedure described by Han and Choi (1996) was used. Briefly, LNCaP cells were incubated with 1 *μ*M ATRA for 24 h to induce the CYP26 enzyme. The cells were trypsinised and rinsed with phosphate-buffered saline (PBS) (Life Technologies, Grand Island, NY, USA) by centrifugation for 5 min at 5000 × **g** at room temperature. The cells were resuspended in homogenate buffer (0.5 M sucrose, 10 mM Tris-Cl (pH 7.4), 1 mM ethylenediamine tetraacetate (EDTA), 1 mM phenylmethylsulfonylfluoride, 0.1 *μ*g ml^−1^ leupeptin, and 0.04 U ml^−1^ aprotonin), homogenised, and then centrifuged at 9000 × **g** for 10 min at 4°C. The supernatant was then centrifuged at 100 000 × **g** for 45 min at 4°C. The pellet was resuspended in storage buffer (0.25 M sucrose, 10 mM Tris-Cl (pH 7.4), 1 mM EDTA, 1 mM phenylmethylsulfonyl-fluoride, 0.1 *μ*g ml^−1^ leupeptin, and 0.04 U ml^−1^ aprotonin) and stored at −20°C.

### Cellular microsomal CYP26 assay

The procedure for the hepatic microsomal enzyme assay (for ATRA 4-hydroxylases) was used where 100 *μ*l of cellular CYP26 microsomes (500 *μ*g ml^−1^ dissolved in storage buffer) was substituted for 100 *μ*l of hepatic microsomes. The retinoid products were extracted, processed, and analysed by HPLC as previously described (Patel *et al*, 2004).

### Cellular CYP26 assay

As modified from a procedure described by Wouters *et al* (1992), approximately 10^6^ LNCaP cells were incubated with 1 *μ*M ATRA for 24 h to induce the CYP26 enzyme. Following ATRA treatment, the LNCaP cells were incubated with 0.1 or 0.8 *μ*M [11,12-^3^H]-ATRA (PerkinElmer Life and Analytical Sciences, Inc., Boston, MA, USA) for 5 h. Following the 5 h incubation, the medium was collected and the cells were trypsinised and collected. The retinoid products were extracted, processed, and analysed by HPLC as previously described (Patel *et al*, 2004).

### WST-1 cell viability assay

To measure cell viability, 24-well plates were coated with a 0.05% poly-L-lysine solution for 30 min. The wells were then washed with sterilised dH_2_O. LNCaP cells (1 × 10^4^) were seeded in the plates and maintained in RPMI 1640 medium (Gibco-Invitrogen, Carlsbad, CA, USA). The cells were allowed to attach for 36 h. After attachment, fresh media was added and the cells were treated with a concentration range of either ATRA or each of the RAMBAs for 6 days. The media was changed every 3 days. On the day the assay was performed, the media was removed and 1 ml of a stock solution of WST-1 (4-[3-(4-iodophenyl)-2-(4-nitrophenyl)-2H-5-tetrazolio]-1,3-benzene disulfonate) cell proliferation reagent (Boehringer Mannheim, Indianapolis, IN, USA), diluted 1 : 10 in RPMI 1640 medium (without FBS), was added to each well. The plates were incubated at 37°C for 3 h. The media was then removed and 500 *μ*l of DMSO was added and agitated vigorously for 5 min. The slightly red tetrazolium salt WST-1 is reduced to a dark red, water-soluble formazan product by mitochondrial dehydrogenase from living cells, which gives absorbance at a wavelength of 450 nm.

### Differentiation assay (Western-blot analysis of cytokeratins 8/18)

LNCaP cells were incubated in the RPMI 1640 medium (Gibco-Invitrogen, Carlsbad, CA, USA) preparation containing 1 *μ*M ATRA alone or in combination with 1 *μ*M of each of the RAMBAs for 6 days. LNCaP cells were scraped with 1.5 ml of PBS. The cells were collected by centrifugation, resuspended in ice-cold cell lysis buffer (0.1 M Tris-HCl, 0.5% Triton X-100, and protease inhibitor cocktail (Boehringer, Indianapolis, IN, USA)), and sonicated. The homogenates were incubated on ice for 30 min, subjected to centrifugation at 13 000 × **g** for 30 min, and the supernatants were separated and stored at −80°C until use. Protein concentrations were determined by the Bradford method using a Bio-Rad kit (Hercules, CA, USA). A measure of 50 *μ*g of cell lysate was subjected to 10% sodium dodecyl sulfate-polyacrylamide gel electrophoresis (SDS–PAGE) using the Mini-PROTEAN3 electrophoresis module assembly (Bio-Rad, Hercules, CA, USA) at 60 V at room temperature. The separated lysates on 10% SDS–PAGE gel were transferred onto Hybond ECL nitrocellulose membrane (Amersham, Arlington Heights, IL, USA) overnight at 20 V at 4°C. Western-blot analysis was performed on the nitrocellulose membrane. The membrane was blocked for 1 h in 10% nonfat dried milk and PBS containing 0.5% Tween 20 (PBS-T) at room temperature. Following washing in PBS-T, the membrane was incubated with mouse monoclonal IgG antibody to cytokeratin 8/18 or *β*-actin (Santa Cruz Biotechnology, Santa Cruz, CA, USA and Oncogene Research Products, Boston, MA, USA, respectively) dissolved in 10% nonfat dried milk (1 : 5000 or 1 : 10 000), respectively) for l h at room temperature. Following washing, the membrane was incubated with horseradish peroxidase-linked anti-mouse IgG antibody (Amersham, Arlington Heights, IL, USA and Oncogene Research Products, Boston, MA, USA, respectively) dissolved in 10% nonfat dried milk (1 : 1000) for 1 h at room temperature. The membrane was incubated in 4 ml ECL Western Blotting Analysis System (Amersham, Arlington Heights, IL, USA) for 1 min at room temperature. The membrane was resolved on chemiluminescence film (Amersham Hyperfilm High Performance chemiluminescence film, Arlington Heights, IL, USA) and the film was developed using an X-ray developer (M35A X-OMAT Processor, Eastman Kodak Company, Rochester, NY, USA). The intensity of the bands on film was analysed using ImageQuant 5.0 software (Amersham, Arlington Heights, IL, USA). The band intensity corresponds to the level of protein expression of cytokeratin 8/18, which is shown to be expressed in differentiated cells of epithelial origin (Peehl *et al*, 1993; Hsieh *et al*, 1995).

### Western-blot analysis of Bad and Bcl-2

The Western-blot protocol described for cytokeratins 8/18 was followed with some exceptions. Cell lysates (50 *μ*g) were separated on a 15% SDS–PAGE gel. All washing was performed using TBS containing 1% Tween 20 (TBS-T). Blocking was performed using 5% milk in TBS-T. Incubations were performed using rabbit monoclonal IgG antibody to Bad and Bcl-2 (1 : 1000), as well as horseradish peroxidase-linked anti-rabbit IgG antibodies (1 : 2000) (Cell Signaling Technology, Beverly, MA, USA).

### TUNEL (terminal deoxynucleotidyl transferase-mediated dUTP nick end labelling) assay

A total of 4000 LNCaP cells were plated on each well of an eight-well Lab-Tek II® chamber slide (Nalge Nunc International, Naperville, IL, USA). The LNCaP cells were then treated with either 1 or 5 *μ*M of ATRA or RAMBA for 6 days. Cells were then fixed in 4% paraformaldehyde solution in dH_2_O. The cells were then processed and stained with FITC (fluorescein isothiocyanate) according to kit instructions (*In situ* cell death detection kit, AP, Roche Diagnostics Corp., Indianapolis, IN, USA). The FITC-stained cells are then mounted with VectorShield® mounting medium for fluorescence with DAPI (4′,6-diamidine-2′-phenylindole dihydrochloride) nuclear stain (Vector Laboratories, Inc., Burlingame, CA, USA) and covered with ApopTag® plastic coverslips (Intergen Company, Purchase, NY, USA). The slides were then examined under fluorescence microscopy (Eclipse E400, Nikon, Inc., Melville, NY, USA) and counted using SPOT Advance® 3.5 software (Diagnostic Instruments, Inc., Sterling Heights, MI, USA).

### Cell-cycle analysis

LNCaP cells were treated with 1 or 5 *μ*M of ATRA or each of the RAMBAs for 6 days. The cells were trypsinised and washed twice in PBS (Life Technologies, Grand Island, NY, USA). The cells were fixed by adding 70% ethanol and stored at −20°C until staining. The cells were washed twice in PBS. A volume of 1 ml of a propidium iodide (PI) solution (50 *μ*g ml^−1^ in PBS) and 500 *μ*l of an RNase stock solution (100 *μ*g ml^−1^) were added to the cells and incubated for 1 h in the dark. Approximately 10^4^ stained cells were then analysed by flow cytometry (Becton Dickenson FACScan, Franklin Lakes, NJ, USA). The percentages of cells in G_0_/G_1_, S, and G_2_/M phases of the cell cycle as well as percentage of apoptotic cells (sub G_0_/G_1_) were determined using MODFIT LT software (Verity Software House, San Jose, CA, USA). The percentages of cells in G_0_/G_1_, S, and G_2_/M phases of the cell cycle were obtained from diploid cells in the sample and the percentage of apoptotic cells (sub G_0_/G_1_) was obtained from total cells minus the debris.

### *In vivo* antitumour studies (LNCaP human prostate carcinoma xenograph mice model)

All animal studies were performed according to the guidelines and approval of the Animal Care Committee of the University of Maryland School of Medicine, and were consistent with United Kingdom Coordinating Committee on Cancer Research guidelines for the welfare of animals in experimental neoplasia. The procedure was modified from Grigoryev *et al* (1999). Briefly, LNCaP cells were trypsinised, counted, and suspended in Matrigel (2 × 10^7^ cells ml^−1^) (Fisher Scientific International, Inc., Hampton, NH, USA). Male SCID mice of 4–6 weeks of age were obtained from the National Cancer Institute (Frederick, MD, USA). Each mouse was inoculated s.c. with 0.1 ml of the cell suspension at two sites in the flank. The size of the tumours was determined by measuring the tumour volumes using calipers. Tumour volumes were calculated using the formula *V*=4/3 × π × *r*_1_^2^ × *r*_2_(*r*_1_<*r*_2_). The tumours were allowed to develop to approximately 100 mm^3^ before treatment. The mice were treated with 10 mg kg^−1^ (which is equivalent to 0.033 mmol kg^−1^) of ATRA or an equivalent dose to 0.033 mmol kg^−1^ of each RAMBA (VN/14-1, VN/50-1, VN/66-1, and VN/69-1) dissolved in hydroxypropyl-*β*-cellulose (HPC, 0.3% in saline). Tumour volumes were measured twice weekly and weights of animals were taken weekly after the initiation of treatment. The mice were subjected to 6 weeks of treatment. The mice were then killed and the tumours were excised, weighed, and stored at −80°C until analysis. Additionally, the plasma was collected with 50 IU of heparin and stored at −80°C until analysis.

### Statistical analysis

One-way analysis of variance (ANOVA) with post test, which was the Bonferroni multiple comparisons test, was performed to compare all pairs of treatment groups using InStat® 3 (GraphPad Software, Inc., San Diego, CA, USA). A *P*<0.05 was considered significant.

## RESULTS

### ATRA metabolism is inducible in LNCaP cells but not in PC3 and DU145 cells

To assess the induction of ATRA metabolism in LNCaP, PC-3 and DU145 prostate cells, these cells were pre-incubated with 1 *μ*M ATRA for various time points from 4 to 48 h and then further incubated with 0.8 *μ*M [11,12-^3^H]-ATRA for 5 h to measure ATRA metabolism. Our results show that LNCaP cells were induced by ATRA and were able to metabolise [11,12-^3^H]-ATRA to more polar products from the 4 to 48 h time points with maximum metabolism occurring at 36 h (Figure 2). PC-3 cells did not show any metabolism of [11,12-^3^H]-ATRA from these time points (Figure 2) and also up to 9 days (data not shown). DU145 cells were able to metabolise [11,12-^3^H]-ATRA from the 4 to 48 h time points (Figure 2); however, this metabolism was not enhanced despite ATRA treatment. As only the LNCaP cell line was able to metabolise ATRA, it was utilised in subsequent cellular assays.

### Inhibition of ATRA metabolism in intact LNCaP cells (cellular assay)

To assess the ability of our novel RAMBA to inhibit ATRA metabolism in intact cells, we used VN/14-1, VN/50-1, VN/66-1, and VN/69-1 to evaluate their inhibitory potencies in LNCaP cells. Human LNCaP carcinoma cells cultured under control conditions are unable to metabolise ATRA into more polar metabolites (data not shown). However, after pretreatment with 1 *μ*M ATRA for 12–15 h, the cells show extensive ATRA metabolism (Figure 3A), converting ATRA into highly polar metabolites (HPM, retention time, *R*_t_=3–6 min), and prominent metabolites of medium polarity (MMP, *R*_t_=8–12 min), including 4-oxo and 4-hydroxy-ATRA. ATRA metabolism is inhibited dose dependently by VN/14-1 (Figures 3B–D). Identical results were also obtained with the other three compounds tested. The IC_50_ values for these compounds were determined from dose–response curves and are presented in Table 1. The compounds inhibited intracellular ATRA metabolism with decreasing activity in the order: VN/14-1>VN/66-1>VN/50-1=VN/69-1. Compound VN/14-1 was the most active with an IC_50_ value of 6.5±1.5 nM. VN/50-1 also inhibited ATRA metabolism of microsomes prepared from T47D cells previously exposed to ATRA with an IC_50_ value of 7.0±1.2 nM, which is of about a 13-fold higher potency compared to the cellular IC_50_ value. Although the other inhibitors were not evaluated using microsomal preparations, it is likely that they may exhibit similar higher potencies.

### Effects of the RAMBAs alone and in combination with ATRA on LNCaP cell growth

Given the retinoidal nature of our RAMBAs it seemed logical to investigate their effects on the growth of LNCaP cancer cells. The antiproliferative effect of ATRA was also studied for comparison using a WST assay. Continuous exposure of LNCaP cells to various doses of the RAMBAs and ATRA for 6 days led to dose-dependent inhibition of cell growth as shown in Figure 4. The calculated IC_50_ values (defined as the concentration of compounds required to inhibit cell growth by 50%) from these dose–response curves are presented in Table 1 and show that all RAMBAs are modest inhibitors (IC_50_=4.5–10.0 *μ*M) of LNCaP cell proliferation and comparable to the potency of ATRA (IC_50_=4.3 *μ*M).

The ability of each of these RAMBAs to enhance the antiproliferative activity of ATRA in LNCaP cells was also studied. ATRA inhibits LNCaP cell proliferation in a concentration-dependent manner (Figure 4) with a calculated IC_50_ value of 4.3 *μ*M (Table 1). For studies of effects of combination of RAMBAs with ATRA, we used low doses (1 *μ*M each, doses that exhibited low [<10%] antiproliferative effects) of RAMBAs. All RAMBAs, that is, VN/14-1, VN/50-1, VN/66-1, VN/69-1 each in combination with ATRA, significantly enhance the antiproliferative activity of ATRA, by 47-, 60-, 70-, 65-fold, respectively (for VN/14-1, IC_50_ from 10.0 to 0.092 *μ*M; for VN/50-1, IC_50_ from 6.0 to 0.071 *μ*M; for VN/66-1, IC_50_ from 4.5 to 0.061 *μ*M; and for VN/69-1, IC_50_ from 5.0 to 0.066 *μ*M) (Figure 4 and Table 1). These enhancements of ATRA activity are considered synergistic because the growth inhibitory effects were each significantly greater than the predicted values.

### Effects of the RAMBAs alone and in combination with ATRA on LNCaP cell differentiation

Cytokeratins 8/18 are present in the differentiated luminal epithelia in prostate and an increase in their content is considered to indicate differentiation (Owens and Lane, 2003). To determine the effects of these retinoids on cell differentiation, LNCaP cells were incubated with various concentrations of ATRA or RAMBA for 6 days; cell lysates were prepared and subjected to SDS–PAGE gel electrophoresis. Increase in cytokeratin 8/18 expression was used as the marker for differentiation (Peehl *et al*, 1993; Hsieh *et al*, 1995). There was a concentration-dependent increase in the expression of cytokeratin 8/18 in LNCaP cells treated with ATRA, VN/14-1, VN/50-1, VN/66-1, and VN/69-1 (data not shown) and also a significant increase in the cytokeratin 8/18 expression for 5 and 10 *μ*M treatment for ATRA and all RAMBAs (*P*<0.05) compared to control (data not shown). This increase in cytokeratin 8/18 expression in LNCaP cells treated with ATRA and the RAMBAs is indicative of differentiation.

For combination studies, LNCaP cells were incubated with 1 *μ*M ATRA, alone and in combination with 1 *μ*M of RAMBA for 6 days. Our results are summarised in Figure 5 and show significantly increase in the expression of cytokeratin 8/18 by 1.18 to 1.38 -fold above control (1±0.07, *P*<0.05). ATRA (1 *μ*M) in combination with 1 *μ*M of each of the RAMBAs, except for VN/69-1, also significantly increased the expression of cytokeratin 8/18 by 1.32–1.59-folds above control [1±0.07, (*P*<0.05)]. Thus, most RAMBAs enhanced the prodifferentiation activity of ATRA in the LNCaP cells.

### TUNEL analysis of LNCaP cells treated with ATRA, 4-HPR, and RAMBAs

One of the possible mechanisms underlying the observed effect of ATRA and the RAMBAs on LNCaP cell viability is the direct induction of apoptosis. To test this hypothesis, TUNEL assays were performed following treatment of cells with the various agents. LNCaP cells treated with 1 or 5 *μ*M ATRA, 4-HPR, or RAMBA for 6 days were processed for TUNEL staining, and viewed under fluorescence microscopy to examine the presence of nicked DNA that are hallmarks of apoptosis. Pictures obtained from the staining of control, ATRA, and VN/69-1 are shown in Figure 6A and results from the TUNEL analysis for all the test compounds are summarised in Figure 6B. Generally, there was a concentration-dependent induction of apoptosis in LNCaP cells treated with 1 or 5 *μ*M ATRA, 4-HPR, VN/14-1, VN/50-1, VN/66-1, and VN/69-1. Treatment with the most potent RAMBAs, VN/14-1 and VN/50-1 (5 *μ*M), yielded a percentage of apoptotic cells of 17.03 and 17.03%, respectively (Figure 6B).

### Effects of ATRA, 4-HPR, and RAMBAs on expressions of Bad and Bcl-2

Bcl-2 is an antiapoptotic protein and acts through inhibition of mitochondrial cytochrome *c* release (Murphy *et al*, 2000). Bad is pro-apoptotic and acts by displacing Bax from binding to Bcl-2 (Yang *et al*, 1995; Zha *et al*, 1996). Bax then dissipates the mitochrondrial membrane releasing cytochrome *c* which leads to apoptosis (Rosse *et al*, 1998). 4-HPR was used as a reference compound as it was well established to induce apoptosis (Fontana and Rishi, 2002). We observed a significant increase in Bad expression for 5 and 10 *μ*M treatment with ATRA and the RAMBAs compared with control (*P*<0.05), with the exception of 4-HPR, where all treatment concentrations of 4-HPR (0.1, 1 and 5 *μ*M) were significant (*P*<0.05) and VN/69-1, where treatments with 1, 5, and 10 *μ*M VN/69-1 were significant (*P*<0.05) (Figure 7). Our results show that there was a concentration-dependent increase in Bad expression in LNCaP cells treated with all of the tested agents. Although there was a concentration-dependent decrease in the expression of Bcl-2 in LNCaP cells treated with ATRA and VN/14-1, treatments with 4-HPR, VN/50-1, VN/66-1, and VN/69-1, however, did not effect Bcl-2 expression (data not shown).

### Cell-cycle analysis of LNCaP cells treated with ATRA, 4-HPR, and RAMBAs

Owing to the antiproliferative activities of these agents, it was of interest to determine their effects on cell-cycle distribution and apoptosis. LNCaP cells were treated with 1 or 5 *μ*M ATRA, 4-HPR, or RAMBA for 6 days. However, only the cell-cycle analysis of LNCaP cells treated with 5 *μ*M of the agents are shown in Figure 8 and also summarised in Table 2. With these treatments, there was no change in the percentage of cells in the G_0_/G_1_ phase (except for ATRA treatment), a decrease in the percentage of cells in the S phase, and an increase in the percentage of cells in the G_2_/M phase (a G2/M cell arrest, Table 2). There was also an increase in the percentage of cell in the sub-G1 phase that indicates apoptotsis in LNCaP cells treated with these agents.

### The effects of ATRA and RAMBAs in LNCaP xenograph SCID mouse model

We next tested the effects of our RAMBAs on growth inhibition of LNCaP tumour xenographs. Male SCID mice bearing LNCaP tumour xenographs (approximately 100 mm^3^) were grouped and treated once daily with 0.033 mmol kg^−1^ each of ATRA or RAMBAs for the indicated time periods. As treatment with VN/50-1 was very toxic to the mice, experiment with this cohort was terminated. Tumour growth in the group receiving VN/14-1 was not significantly different from that observed in the vehicle control group (Figure 9). Mice receiving VN/66-1 or VN/69-1 had 44 and 47%, respectively, reduction in tumour growth compared to control (*P*<0.05), whereas treatment with ATRA reduced tumour growth by 75% relative to control (*P*<0.05) (Figure 9). During the study, all mice were weighed twice a week. Whereas the body weights of mice in the RAMBAs-treated groups were not altered, weights of mice in the ATRA-treated group were significantly (∼25%) reduced compared to control (data not shown).

## DISCUSSIONS

The prompt emergence of resistance to ATRA therapy in oncology and dermatology is a major concern in the development of ATRA (Miller, 1998; Njar, 2002, 2006). Agents that are able to inhibit ATRA metabolism may be used alone or in combination with low doses of ATRA or other therapeutic agents for the treatment of a variety of cancers and dermatological diseases. Interest in the development of structurally diverse agents as RAMBAs has increased over the last 5 years. We have developed several atypical RAMBAs with retinoidal scaffolds that possess unique multiple biological activities (Patel *et al*, 2004, 2006; Belosay *et al*, 2005; Njar, 2006). In this study, we have investigated the effects and mechanisms of action of several of these RAMBAs in human prostate LNCaP carcinoma cells.

Han and Choi (1996) were able to induce ATRA 4-hydroxylase in T47D cells and suggested that ATRA induces its own metabolism through a negative feedback mechanism. The enzyme activities induced by ATRA appear to be regulated at the level of transcription. Our results showed that only LNCaP cells were able to metabolise ATRA through induction of CYP26 (Figure 2). PC3 cells did not metabolise ATRA and the metabolism of ATRA did not exceed basal levels despite induction of CYP26 in DU145 cells. These results suggest that hormone-dependent cells (LNCaP) are able to metabolise ATRA following induction of CYP26 and that hormone-independent cells (PC3 and DU145) are not. To the best of our knowledge, this appears to be the first report of induction of ATRA 4-hydroxylase (CYP26) in LNCaP cells. The lack of ATRA-induced CYP26 in both PC3 and DU145 cells may be due to the loss of RARs in these cell lines. Several investigators have reported that the loss of RAR signaling in many epithelial tumours is driven by loss of expression of RARs (Campbell *et al*, 1998; Zhang, 2002).

**Figure 1:**
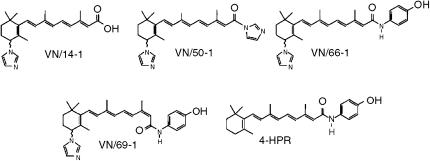
Chemical structures of RAMBAs, VN/14-1, VN/50-1, VN/66-1, and VN/69-1 and 4-HPR.

**Figure 2:**
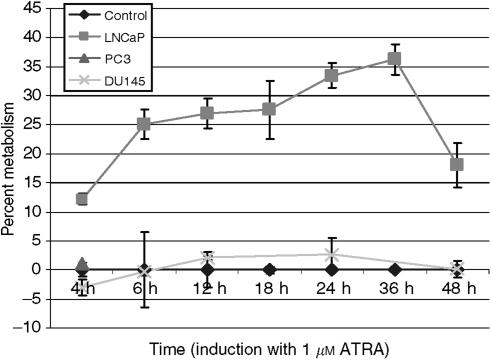
Time course of induction of CYP26 in prostate cancer cells. LNCaP, PC3, and DU145 cells were pre-incubated with 1 *μ*M ATRA for various time periods to induce the CYP26 enzyme. Cells were then isolated and incubated at 37°C for 5 h with 0.8 *μ*M [11,12-^3^H]-ATRA. Retinoids were extracted, and metabolites analysed by HPLC as described in Materials and Methods. Metabolic activity was calculated as percentage of polar ATRA metabolites of total radioactivity. The metabolic activity of induced LNCaP, PC3, and DU145 cells was then divided by the metabolic activity of the respective un-induced cells (percent metabolism). The percent metabolism of each un-induced cell line was set to the *x*-axis (Control) and the percent metabolism of the induced cell lines was plotted accordingly. The experiments were performed thrice.

**Figure 3:**
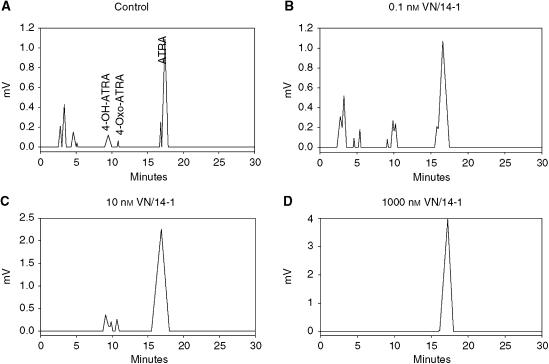
Inhibition of ATRA metabolism by VN/14-1 in intact LNCaP cells. Human LNCaP prostate cancer cells were cultured under basal conditions (data not shown) or pretreated with 1 *μ*M ATRA (**A**–**D**). Thereafter, cells were washed, and incubated with 0.1 *μ*M [11,12-^3^H]-ATRA, either in the absence (**A**) or presence (**B**–**D**) of VN/14-1 at concentrations of 0.1, 10, and 1000 nM, respectively. The cells and media were collected, extracted and analysed by reverse phase HPLC as described in Materials and Methods. The experiments were performed twice.

Based on the IC_50_ values of the RAMBAs in hamster hepatic microsomes that were obtained previously (Patel *et al*, 2004), representative RAMBAs (VN/14-1, VN/50-1, VN/66-1, and VN/69-1) (Figure 1) were studied further in LNCaP cells. In intact LNCaP cells, these compounds potently inhibit ATRA metabolism (Figure 3C–D and Table 1). The IC_50_ values obtained for VN/14-1, VN/66-1, and VN/69-1 were comparable to the values seen in cellular MCF7 and T47D breast cancer cells that were determined previously (Patel *et al*, 2004). However, there was a discrepancy in the IC_50_ value for VN/50-1 (10.0 nM in T47D cells *vs* 90.0 nM in LNCaP cells). This difference may be due to the ability of VN/50-1 to penetrate the cell membranes of the LNCaP PCA cells. Similar results have recently been reported for farnesol derivatives that are weak inhibitors of ATRA metabolism in human head and neck squamous cell carcinoma (AMC-HN-6) cells and their microsomal preparations (Kim *et al*, 2001).

**Figure 4:**
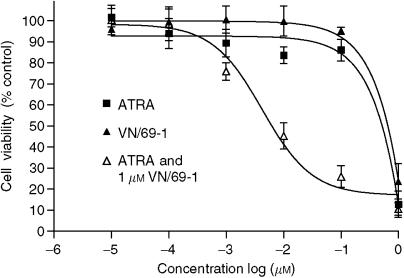
Antiproliferative effects of ATRA or VN/69-1 alone and ATRA in combination with VN/69-1 (1 *μ*M). LNCaP cells were incubated with various concentrations of ATRA, the RAMBAs alone and in combination. The MTT assay was performed. The plot represents the percentage of viable cells *vs* the concentration of agents used. The IC_50_ value was determined as the concentration of agents that inhibited the viability of LNCaP cells by 50%. Similar results were obtained for VN/14-1, VN/50-1, VN/66-1, and 4-HPR.

**Figure 5:**
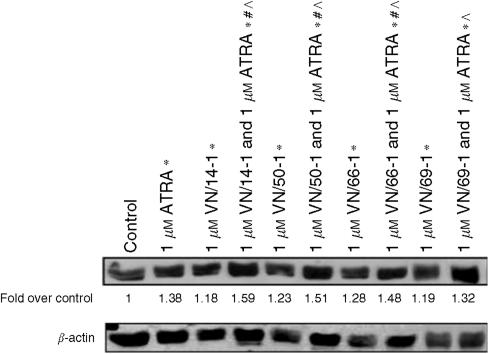
Effects of ATRA and RAMBAs alone and in combination on levels of cytokeratin 18 (a differentiation marker) in human prostate LNCaP cells. Cells were incubated with ATRA or RAMBAs alone or in combinations for 6 days. Lysates were subjected to SDS–PAGE and Western blotting. Membranes were probed with cytokeratin 18 antibody, and intensities of bands were analysed by densitometry. Groups labelled with ^*^ are significantly different from control (*P*<0.05). Groups labelled with # are significantly different from ATRA alone (*P*<0.05). Groups labelled with ^ are significantly different between combination RAMBA with ATRA compared to the corresponding RAMBA alone (*P*<0.05).

**Figure 6:**
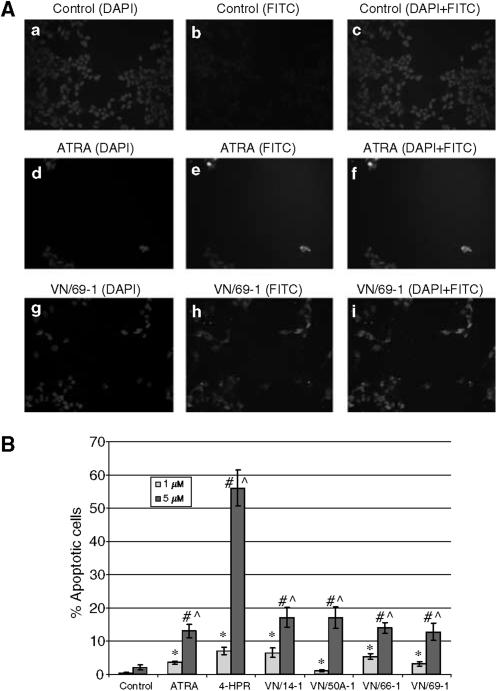
(**A**) Apoptosis in LNCaP cells determined by TUNEL and analysed by fluorescence microscopy. LNCaP cells were treated with 5 *μ*M ATRA or RAMBA for 6 days. Cells were then fixed, stained, mounted, and examined under fluorescence microscopy. Control is shown in **a**, **b**, and **c**. ATRA (5 *μ*M) is shown in **d**, **e**, and **f**. VN/69-1 (5 *μ*M) is shown in **g**, **h**, and **i**. Nuclear DAPI staining is shown in **a**, **d**, and **g**. FITC staining for nicked DNA is shown in **b**, **e**, and **h**. Combined DAPI and FITC staining is shown in **c**, **f**, and **i**. (**B**) TUNEL analysis of LNCaP cells treated with ATRA, 4-HPR, or RAMBAs. LNCaP cells were incubated with either 1 or 5 *μ*M of ATRA, 4-HPR, or a RAMBA for 6 days. LNCaP cells were then fixed, stained, mounted, and analysed by fluorescence microscopy. The number of apoptotic cells and total number of cells were counted in each of the five fields for each treatment and the percentage of apoptotic cells was calculated and plotted. Treatment with 1 *μ*M drugs significantly increased from its control (^*^) (*P*<0.05). Treatment with 5 *μ*M drugs significantly increased from its control (#) (*P*<0.05). Treatment with 5 *μ*M of drugs significantly increased from 1 *μ*M of the same drug (^) (*P*<0.05).

**Figure 7:**
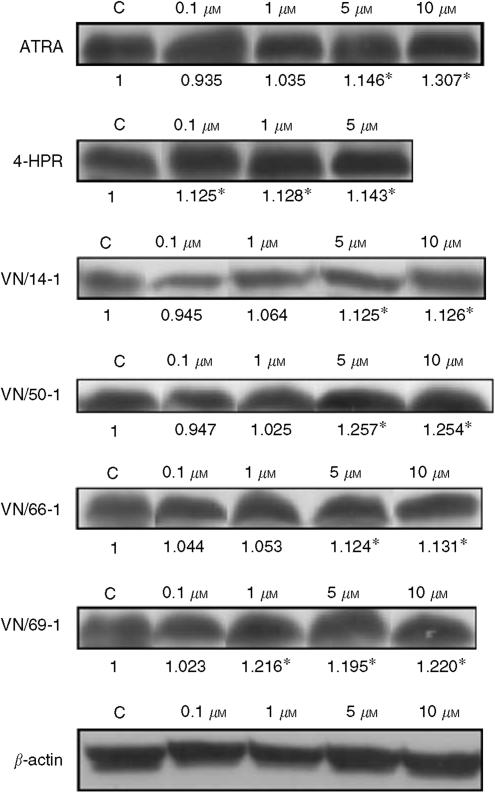
Western immunobloting of whole-cell lysates of treated LNCaP cells for expression of Bad. Cell lysates were used as described in the differentiation assay. Bad is a proapoptotic protein. The Western blots of Bad expressions are shown. The lanes are labelled above the blots and the expression of Bad is expressed as fold over control as determined by densitometry (below the blot). There was a significant difference in groups labelled with ^*^ compared to control (*P*<0.05).

**Figure 8:**
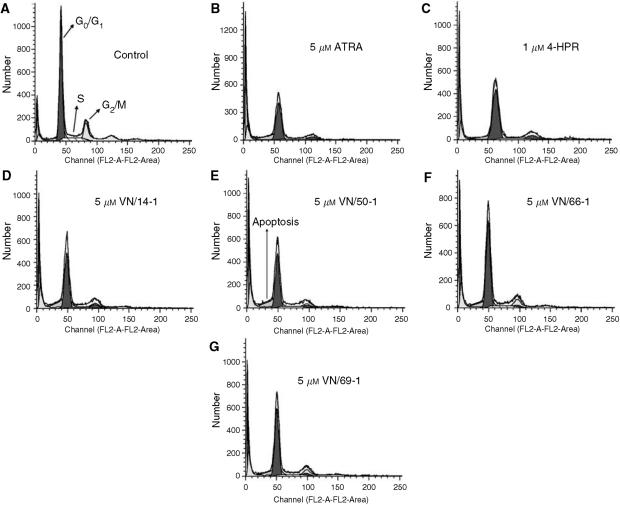
Cell-cycle analysis of LNCaP cells treated with 1 or 5 *μ*M ATRA, 4-HPR or RAMBAs. LNCaP cells were incubated with either 1 or 5 *μ*M ATRA, 4-HPR, or RAMBA for 6 days. LNCaP cells were then fixed, stained with propidium iodide, and analysed by FACScan. Histograms of the FACScan analysis from control (**A**), ATRA (**B**), 4-HPR (**C**), VN/14-1 (**D**), VN/50A-1 (**E**), VN/66-1 (**F**), and VN/69-1 (**G**) are shown.

In LNCaP cell proliferation experiments, each of the RAMBAs tested enhanced the antiproliferative activity of ATRA (Figure 4 and Table 1). The antiproliferative effects of ATRA was enhanced by 47- to 70-fold with the addition of 1 *μ*M VN/14-1, VN/50-1, VN/66-1, or VN/69-1. Concentrations of RAMBAs effective in enhancing the antiproliferative activity of ATRA are themselves unable to significantly decrease LNCaP cell proliferation. Thus, these data support the hypothesis that our compounds enhance the biological activity of ATRA through inhibition of ATRA metabolism. These results are identical with those previously observed in human breast MCF-7 cancer cells (Patel *et al*, 2004), but are superior to the effects observed with R116010 in human breast T47D cancer cells (Van Heusden *et al*, 2002). In the latter study, R116010, at a concentration of 1 *μ*M, enhanced the antiproliferative activity of ATRA only by threefold. As expected, our RAMBAs also inhibited the growth of LNCaP cells in a dose-dependent manner, with IC_50_ values ranging from 4.3 to 10 *μ*M (Figure 4 and Table 1), which correlates with their intrinsic retinoidal antiproliferative activities.

Having established the *in vitro* antiproliferative activities of these novel compounds alone and in combination with ATRA, we set out to investigate possible molecular mechanisms that might be involved using established procedures. On the basis of our previous studies and knowledge that these RAMBAs possess both intrinsic retinoid-like activities and also ATRA-mimetic effects (Patel *et al*, 2004), we assessed their effects on cell differentiation, apoptosis and cell cycle.

Indeed, this study presents evidence that our RAMBAs induce differentiation (via upregulation of cytokeratin 8/18 expression) and also significantly enhance ATRA-induced differentiation in the LNCaP cells. We have previously shown that with the exception of VN/14-1, the other three RAMBAs were weak ligands and activators of the RAR*α*. As it is believed that only ligands that activate RAR*α* induce cell differentiation (Bollag *et al*, 1997), the present data suggest that induction of differentiation can also occur via RAR*α*-independent pathway. The ability of the RAMBAs to enhance ATRA-induced differentiation may be attributed to intracellular inhibition of ATRA metabolism with concomitant accumulation of cellular ATRA.

In the normal prostate, there is a balance between cell proliferation and cell death (Denmeade *et al*, 1996), while in PCA, the proliferative rate remains relatively low, but there is less apoptosis (Berges *et al*, 1995). Our results here demonstrate that RAMBAs were able to induce apoptosis in LNCaP cells as determined by the TUNEL assay. Induction of apoptosis appears to be associated with upregulation of the proapoptotic protein Bad and reduction or no change in the levels of antiapoptotic Bcl-2. Furthermore, cell-cycle analysis revealed that these agents significantly induce arrest of cell in the G_2_/M phase. It should be stated that there is a major checkpoint in the G_2_/M phase of the cell cycle where cells arrested in the G_2_/M phase can undergo apoptosis if optimal conditions are not met (Kastan and Bartek, 2004). This scenario may also be operational in this study. Therefore, our findings that our RAMBAs cause G_2_/M arrest and induce apoptosis in the LNCaP cells offer a potential new therapeutic option for PCA therapy.

**Figure 9:**
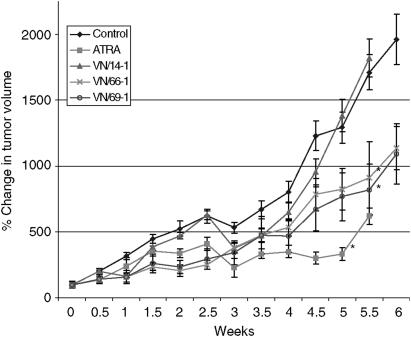
The effect of ATRA, VN/14-1, VN/66-1 on LNCaP tumour volumes in male SCID mice. Male SCID mice were inoculated s.c. with LNCaP cells suspended in Matrigel at two sites in the flank. The sizes of the tumours were determined by measuring the tumour volumes using calipers. Tumour volumes were calculated using the formula V=4/3 × *π* × *r*_1_^2^ × *r*_2_ (*r*_1_<*r*_2_). The tumours were allowed to develop to 100 mm^3^ before treatment. The mice were treated with an equivalent dose to 0.033 mmol kg^−1^ of ATRA, VN/14-1, VN/66-1 or VN/69-1. The vehicle control was hydroxypropyl-*β*-cyclodextrin (HPC) in saline. (^*^) indicated that these treatment groups were significantly different from vehicle control (*P*<0.05).

The marked effects that our RAMBAs had on cell proliferation led to *in vivo* studies. Our *in vivo* studies showed that VN/14-1 was ineffective in this model as it did not cause inhibition of tumour growth (Figure 9). VN/50-1 was toxic to the animals and its antitumour efficacy could not be assessed. The lack of antitumour efficacy of VN/14-1 in the LNCaP PCA model is rather puzzelling because the compound has been shown to possess excellent antitumour efficacy in the MCF-7 human breast cancer model (Patel *et al*, 2004, 2006). Clearly, more studies are required to explain this unexpected finding. In contrast to the effects of VN/14-1, the other two RAMBAs, VN/66-1 and VN/69-1 as well as ATRA exhibited statistically significantly LNCaP tumour growth inhibition. Despite the fact that ATRA exhibited a more potent antitumour activity, the significant decrease (25%) in body weights of animals in this cohort is indicative of toxicity, which is a matter of concern. Although a first generation RAMBA, liarozole has been shown to exhibit potent antitumour efficacy against human androgen-independent PCA tumours, such as PC-3ML-B (Stearns *et al*, 1993), and DU-145 (Smets *et al*, 1994) and several rat PCA tumours (De Coster *et al*, 1992; Dijkman *et al*, 1994); this study appears to be the first report on the antitumour efficacy of RAMBAs against human androgen-dependent LNCaP PCA tumours. Recently, it was demonstrated that a second generation RAMBA, R116010, was able to significantly inhibit the growth of murine TA3-Ha mammary tumours in A/J mice (Van Heusden *et al*, 2002). Given that our RAMBAs exhibited potent *in vitro* antiproliferative activities, their *in vivo* antitumour efficacies are rather disappointing. The reason(s) for these discrepancies are unknown at this time but could be caused by the rapid metabolism of the various RAMBAs to inactive metabolites *in vivo* or the combined effects of RAMBAs and endogenous constituents that, in combination with the RAMBAs exert poor inhibitory effects on tumour growth. Clearly, further *in vivo* studies using other doses of the RAMBAs and modes of administrations are necessary to determine fully the usefulness of these novel compounds in PCA.

In conclusion, we have identified potent inhibitors of ATRA metabolism in intact human LNCaP PCA cells. Inhibition of ATRA metabolism leads to enhanced antiproliferative activity of ATRA both *in vitro* and *in vivo*. This report, to our best knowledge, is the first to demonstrate the induction of cell differentiation, apoptosis and G_2_/M cell cycle arrest by RAMBAs in human PCA cells. Our findings suggest that additional studies to determine the efficacy of our novel RAMBAs in the treatment of human prostate cancer are warranted.

## Figures and Tables

**Table 1:** IC_50_ values of the RAMBAs on cellular CYP26 and LNCaP cell growth inhibition assays

		**Cell growth inhibition^b^**		
		**Agents alone**	**Combination with RAMBAs^c^**	
**Compound**	**Cellular CYP26 assay^a^ IC_50_ (nM)**	**IC_50_ (*μ*M)**	**IC_50_ (*μ*M)**	**Fold enhancement**
ATRA	—	4.3	—	
VN/14-1	6.5±1.5	10.0	0.092^*^	+47
VN/50-1	90.0±10.0	6.0	0.071^*^	+60
VN/66-1	62.5±12.5	4.5	0.061^*^	+70
VN/69-1	90.0±12.5	5.0	0.066^*^	+65

a The cellular CYP26 assay was performed using [11,12-^3^H]-ATRA as a substrate for CYP26 with the addition of various concentrations of each RAMBA. The retinoids were extracted, processed, and analysed by HPLC. Percent metabolism *vs* the concentration of RAMBA used was obtained and graphed. IC_50_ values were determined as the concentration of RAMBA that inhibited the metabolism of [11,12-^3^H]-ATRA by 50%.

b For cell growth inhibition studies, LNCaP cells were incubated with various concentrations of ATRA and the RAMBAs alone and in combination. The WST-1 assay was performed. The plot represents the percentage of viable cells *vs* the concentration of RAMBA used. The IC_50_ value was determined as the concentration of RAMBA that inhibited LNCaP cell viability by 50%.

c 1 *μ*M of each RAMBA was used. Statistical significance was defined at the level of ^*^P<0.05.

**Table 2:** Cell-cycle analysis of LNCaP cells treated with 1 or 5 *μ*M ATRA, 4-HPR or RAMBAs

**Treatment**	**G_0_/G1 (%)**	**S (%)**	**G2/M (%)**	**Sub-G1 (%)**
Control	82.78	15.01	2.20	9.02
ATRA (5 *μ*M)	77.74	9.84	12.42	13.64
4-HPR (1 *μ*M)^a^	79.53	9.18	12.03	7.52
VN/14-1 (5 *μ*M)	82.59	7.26	10.15	17.88
VN/50-1 (5 *μ*M)	81.33	11.38	7.30	17.67
VN/66-1 (5 *μ*M)	83.80	11.14	5.07	14.49
VN/69-1 (5 *μ*M)	82.66	11.14	6.20	13.39

LNCaP cells were incubated with either 1 or 5 *μ*M ATRA, 4-HPR, or RAMBA for 6 days. LNCaP cells were then fixed and stained with propidium iodide. 10^4^ LNCaP cells were analysed by FACScan. The percentages of cells in the G_0_/G_1_, S, and G_2_/M were calculated from diploid cells. The percentage of apoptotic cells (sub G_0_/G_1_) was calculated from the total number of cells minus the debris. The percentages of cells in G_0_/G_1_, S, and G_2_/M phases of the cell cycle as well as percentage of apoptotic cells (sub-G_1_) were determined using MODFIT LT software.

a Treatment with 5 *μ*M 4-HPR resulted in a massive amount of cell death such that no cell-cycle analysis could be performed.
